# Benzoyl Peroxide Formulated Polycarbophil/Carbopol 934P Hydrogel with Selective Antimicrobial Activity, Potentially Beneficial for Treatment and Prevention of Bacterial Vaginosis

**DOI:** 10.1155/2013/909354

**Published:** 2013-12-07

**Authors:** Shiqi Xu, Veronica L. Cavera, Michael A. Rogers, Qingrong Huang, Konstantin Zubovskiy, Michael L. Chikindas

**Affiliations:** ^1^School of Environmental and Biological Sciences, Rutgers State University, 65 Dudley Road, New Brunswick, NJ 08901, USA; ^2^Department of Biochemistry and Microbiology, Rutgers State University, 76 Lipman Drive, New Brunswick, NJ 08901, USA; ^3^Scientelle, LLC, Morristown, NJ 07906, USA; ^4^Bioronus, LLC, Highland Park, NJ 08904, USA

## Abstract

The human vagina is colonized by a variety of indigenous microflora; in healthy individuals the predominant bacterial genus is *Lactobacillus* while those with bacterial vaginosis (BV) carry a variety of anaerobic representatives of the phylum *Actinobacteria*. In this study, we evaluated the antimicrobial activity of benzoyl peroxide (BPO) encapsulated in a hydrogel against *Gardnerella vaginalis*, one of the causative agents of BV, as well as indicating its safety for healthy human lactobacilli. Herein, it is shown that in well diffusion assays *G. vaginalis* is inhibited at 0.01% hydrogel-encapsulated BPO and that the tested *Lactobacillus* spp. can tolerate concentrations of BPO up to 2.5%. In direct contact assays (cells grown in a liquid culture containing hydrogel with 1% BPO or BPO particles), we demonstrated that hydrogels loaded with 1% BPO caused 6-log reduction of *G. vaginalis*. Conversely, three of the tested *Lactobacillus* spp. were not inhibited while *L. acidophilus* growth was slightly delayed. The rheological properties of the hydrogel formulation were probed using oscillation frequency sweep, oscillation shear stress sweep, and shear rate sweep. This shows the gel to be suitable for vaginal application and that the encapsulation of BPO did not alter rheological properties.

## 1. Introduction

The healthy human vagina is colonized by a variety of bacterial species with lactobacilli being a predominant group of microorganisms. The cause and etiopathogenesis of bacterial vaginosis (BV) is still poorly understood; however, it has been extensively shown that when the natural ecology shifts to mainly Gram-negative *Actinobacteria*, microbial disease such as BV can occur [1]. BV affects one in three women in the United States. Initially, infection leads to discomfort and a foul discharge while long-term infection has been correlated with increased risk of pelvic inflammatory disease, sexually transmitted infections, and pregnancy complications including preterm birth [2, 3]. Advanced methodological approaches utilizing qPCR and deep sequencing confirm BV as a multispecies infection [4, 5]. However, *G. vaginalis* is observed in approximately 70% of tested women regardless of whether the individual is considered positive for BV based on the Nugent criteria [5–7].

Current Food and Drug Administration (FDA) approved treatments include nitroimidazoles (i.e., metronidazole) or the lincosamide clindamycin [8]. These antibiotics alter the indigenous microflora by inhibiting both the problematic and healthy bacterial species. *G. vaginalis* and other anaerobic bacteria grow in complex biofilms; therefore, a high dose of antibiotics is required to inhibit their growth. This high-dose requirement causes wide-spectrum microbial elimination and arrests the competitive exclusion capability of *Lactobacillus* leading to increased tolerance of BV-associated microorganisms [9]. Recurrence of resistant BV-associated pathogens is seen in more than 50% of women up to a year following treatment from metronidazole or clindamycin [10, 11]. Although there is no established causation between antibiotic usage and recurrence incidence, it is plausible that the lack of selectivity in available antibiotic treatment options may severely disrupt restoration of normal vaginal microbiota, which could prevent BV recurrence [8]. Lactobacilli are naturally occurring representatives of the vaginal microbiota, beneficiary to overall vaginal health. These bacteria effectively protect the vaginal environment against pathogens by producing natural antimicrobials such as hydrogen peroxide, bacteriocins, and weak organic acids [9, 12]. Usually, healthy vaginal environments are colonized by predominantly one or two species of lactobacilli (generally *L. crispatus* or *L. iners*). Women with recurrent BV infections have been documented to have a wider variety of lactobacilli including but not limited to *L. gasseri*, *L. plantarum*, *L. jensenii*, and *L. vaginalis* [13, 14]. Due to this wide variety of nonpathogenic bacteria, it is imperative to treat the pathogenic microorganisms with a more selective antimicrobial which is harmless to lactobacilli and does not promote resistance in pathogens.

**Figure 1:**
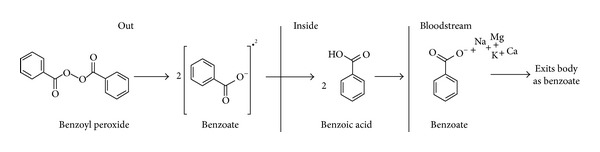
The breakdown of BPO upon contact with epithelium. BPO undergoes a homolytic fission resulting in two benzoate radicals which pass into the bloodstream where it is protonated into benzoic acid then deprotonated. Free cations in the blood will be attracted, but no further modifications will occur while in the body.

BPO is an organic peroxide included on the World Health Organization (WHO) Model Lists of Essential Medicines [15, 16]. It is identified as safe for human use and is found in a variety of products ranging from flour bleaching agents to cleaning products. Further, BPO is often used in topical formulations because it is reasonably stable in gel solutions. Free radical generation from BPO has not been associated with acquired resistance in acne-associated cases [16, 17]. Resistance from BPO usage has not been reported for *G. vaginalis*; moreover, naturally occurring *Lactobacillus* spp. produce hydrogen peroxide which also utilizes a homolytic fission to competitively inhibit other bacteria. When used in topical medicinal or cosmetic formulations, such as in a hydrogel treating acne, BPO undergoes homolytic fission resulting in two benzoate radicals due to the perester nature of the compound. As benzoate passes through the epithelium, a proton is acquired, altering the compound into benzoic acid which diffuses freely into the bloodstream where it is deprotonated due to its low pKa [18]. Free cations in the blood will be attracted to benzoate and the resultant structure is excreted without further alteration (Figure 1).

Antibiotics targeted at vaginal pathogens are administered either orally or intravaginally. The latter requires a delivery system which provides physical contact of the antimicrobial agent with the mucosa. Gels represent one of the most frequently used intravaginal delivery systems due to their acceptability, feasibility, and low cost [19]. This delivery system reduces the bacterial load through the formation of a physical barrier with the vaginal topography which enhances natural defenses of the vaginal environment [12, 20]. The most effective gels also need to be mucoadhesive; this allows for an extended period of contact between the encapsulated drug and the vaginal epithelium while maintaining its properties [21, 22].

Several currently available vaginal hydrogels (Crinone, Replens, RepHresh, Advantage-S, Miphil, etc.) are utilizing bioadhesive properties of polycarbophil which belongs to the poly(acrylic) acid group.

The underlying hypothesis for this study was that the BPO encapsulated in bioadhesive hydrogel would have a selective antibacterial profile required for a sustained treatment effect in BV, making it a good candidate for further development. In this study, we developed a polycarbophil/Carbopol 934P hydrogel with encapsulated BPO. The resulted gel formulation was capable of controlling the BV-associated pathogen *G. vaginalis* while not inhibiting four healthy vaginal microorganisms (*L. acidophilus*, *L. gasseri*, *L. plantarum,* and *L. vaginalis*). This gel is conceptually close to already marketed products with proven safety features, mimics the function of the peroxide-producing lactobacilli, and therefore could be a promising candidate for treatment of BV. The rheological properties of the formulation evaluated *in vitro* indicate that the gel will maintain its structure within the vaginal environment.

## 2. Materials and Methods

### 2.1. Chemical Compounds

Noveon AA-1 polycarbophil was obtained from Lubrizol Advanced Materials, Inc. (Cleveland, OH, USA). Hydrous BPO (74% BPO, 26% water), Carbopol 934P, acetic acid, and sodium acetate were from Spectrum Chemical Manufacturing Corp. (New Brunswick, NJ, USA).

Vaginal fluid simulant (VFS) used in rheological evaluation was composed of (g/L ddH_2_O): 3.51 sodium chloride, 0.222 calcium hydroxide, 3.41 potassium hydroxide, 0.018 bovine serum albumin, 2.0 lactic acid, 1.0 acetic acid, 0.16 glycerol, 0.4 urea, and 5.0 glucose. To adjust pH to 4.2, 6N hydrochloric acid was added [23]. Sodium chloride, bovine serum albumin, lactic acid, and glucose were purchased from Sigma-Aldrich Co (St. Louis, MO, USA). Calcium hydroxide, glycerol, and urea were purchased from Fisher Scientific Inc, (Pittsburgh, PA, USA). Potassium hydroxide and hydrochloric acid were purchased from VWR International LLC (Radnor, PA, USA). Clindamycin (Tokyo Chemical Industry, Tokyo, Japan) was used as a positive control for well diffusion experiments. Aqueous stock solutions of clindamycin were filter-sterilized through 0.2 *μ*m syringe filters (NALGENE, Rochester, NY, USA).

### 2.2. Bacterial Strains, Growth, and Conditions

*G. vaginalis* ATCC 14018 was used as the BV-associated pathogen and was stored at −80°C in Brain Heart Infusion (BHI) medium (Difco, Sparks, MD, USA) supplemented with 3% horse serum (HS) (JRH Biosciences, KS, USA) and 15% (by volume) glycerol. Frozen stocks were cultured on human blood bilayer Tween (HBT) agar (Remel, Lenexa, KS, USA) and grown at 37°C in 5% CO_2_ and 2.5% H_2_ for 48 hours using EZ Anaerobe Container System GasPak (Becton, Dickinson and Co., Sparks, MD, USA). Experiments were performed in Type A Coy Laboratory Vinyl Anaerobic Chamber (Coy Laboratory Products, Grass Lake, MI, USA). The anaerobic conditions are identical to those provided by the EZ Anaerobe Single colonies which were streaked onto HBT plates for the modified agar streak well diffusion assay. All media and agar for *G. vaginalis* were preincubated in the aforementioned anaerobic conditions for 24 hours to remove oxygen-related stress.

*L. vaginalis* ATCC 49540, *L. gasseri* ATCC 33323, *L. plantarum* ATCC 39268, and *L. acidophilus* ATCC 4356 were representative of the normal flora of a healthy vagina [13, 14]. *L. gasseri *and *L. vaginalis *are representative of vaginal flora of healthy women while *L. acidophilus* and *L. plantarum* have been isolated in women with recurrent BV infections. These were selected as they represent a wider net of nonpathogenic bacteria that should not be inhibited to ensure continued health and maintenance of the vaginal environment. They were stored at −80°C in DeMan, Rogosa, and Sharpe (MRS) broth (Oxoid, Hampshire, England) containing 15% glycerol by volume. The cells were plated on 1.5% w/v MRS agar and grown aerobically at 37°C. For experimental procedures, single colonies were inoculated in 20 mL of MRS and grown aerobically for 24 hours with agitation (100 RPM). Cells were subcultured twice before use. For all experiments, 200 *μ*L of the overnight culture was transferred into 20 mL of fresh broth.

### 2.3. Hydrogel Preparation

Base gel was defined as hydrogel without BPO. The base gel is prepared as follows (w/w): polycarbophil/Carbopol 934P were hydrated in double distilled water. Sodium acetate, acetic acid, Carbopol gel, and glycerol were slowly added to polycarbophil gel while stirring. The final concentrations of these components were 2% polycarbophil, 1% Carbopol 934P, 15% glycerol, 0.049% sodium acetate, and 0.038% acetic acid. To elevate pH of base gel from 3.20 to 4.50, 5 M sodium hydroxide solution was used.

BPO particles were evenly dispersed in base gel at predetermined concentrations from 0.01% to 10% in Nasco Whirl-Pak bags (Fisher Scientific Inc., Pittsburgh, PA, USA). The gel was then placed into 50 mL tubes (Becton Dickinson, Franklin Lakes, NJ, USA) and centrifuged at 720 relative centrifugal force (RCF in *g* forces) at ambient temperature for 10 minutes in an Allegra 21R Centrifuge (54180 fixed angle rotor) (Analytical Instrument Brokers, LLC, MN, USA).

### 2.4. Agar Streak and Soft Agar Overlay Well Diffusion Assays

A modified agar streak method described by Waksman and Reilly was used for *G. vaginalis* as the microorganism does not grow well in soft agar [24]. Briefly, colonies isolated from frozen stock were restreaked on preincubated (i.e., kept in the anaerobic environment overnight) HBT plates. To obtain a homogenous lawn of *Lactobacillus* spp., 5 mL of MRS soft (0.7% w/v) agar was inoculated with overnight cultures (10^7^ CFU/mL) and evenly distributed over MRS (1.5% w/v) agar plate. Soft agar overlay plates were dried for approximately one hour in a Purified Class II Safety Cabinet (LabConco Co., Kansas City, MI, USA).

Wells were then aseptically punched through the soft agar overlay and the hard agar with the back of a 1000 *μ*L tip. Each well was filled with 100 *μ*L of gel formulation. Fifty *μ*L of 100 *μ*g/mL clindamycin was used as a positive control. Plates were incubated in an anaerobic jar (Sigma Aldrich, St. Louis, MO, USA) for 24 hours at 37°C. After incubation, zones of inhibition were measured with Vernier calipers (Nova-Tech, Houston, TX, USA). This experiment was performed at least two times in triplicates.

### 2.5. Direct Contact Inhibition Studies

Inhibition studies were conducted using the time kill method as described by Liang et al. [25] with modifications. All bacteria were grown in contact with 5 mL 1% BPO gel, 5 mL base gel, or 0.05 g BPO particles (0.067 g hydrous BPO). BPO particles are defined as the hydrous BPO compound without encapsulation in the base gel. BPO was not dissolved in ethanol or dimethyl sulfoxide (DMSO) as these do not simulate what would be used in an actual product. Each assay included a growth control without a test sample as a negative control. The test sample was placed at the bottom of a 50 mL tube, followed by 40 mL of either BHI + 3% HS or MRS broth. Overnight cultures of *G. vaginalis* were diluted to 10^6^ CFU/mL while the four *Lactobacillus* spp. were diluted to 10^3^ CFU/mL. Cells were grown in direct contact with base gel, BPO gel, or BPO particles and incubated anaerobically at 37°C. At 0-, 1-, 3-, 6-, 9-, 12- and 24-hour time intervals, 300 *μ*L of the culture was taken out for viable cell enumeration by the drop plate counting method as described by Herigstad et al. [26]. The experiment was carried out at least two times in duplicates. *Q* test was performed and *Q*_90%_ was set as rejection level.

### 2.6. Inhibition by BPO Released from the Gel

To avoid direct contact between the targeted cells and the released antimicrobial, the inhibition of *G. vaginalis* by the BPO gel through a 0.45 *μ*m diffusible membrane was tested via a control insert assay. Briefly, an overnight culture of *G. vaginalis* was diluted to 10^6^ CFU/Ml; then 600 *μ*L of the culture dilution was transferred onto the bottom of a 24-well control insert plate (Becton, Dickinson and Co., Bedford, MA, USA). The control inserts were then placed into the wells and 50 *μ*L of 1% BPO gel, base gel, or 0.5 mg BPO particles was placed on the top of each membrane. At the 0-, 1-, 3-, 6-, 9-, 12-, and 24-hour time intervals, 200 *μ*L of culture was removed for enumeration by the drop plating method on HBT agar plates [26]. This experiment was carried out twice in duplicate for a total of 4 replicates. *Q* test was performed and *Q*_90%_ was set as rejection level.

### 2.7. Microbial Growth in pH Adjusted Media

All cultures were grown in aforementioned, standard conditions for 24 hours at which time 200 *μ*L of overnight culture was transferred to 20 mL of pH adjusted media (MRS for *Lactobacillus* spp. and BHI + 3% HS for *G. vaginalis*). Media pH was adjusted to 4.5 (the average pH of all gels) through simple titration using either 0.1 M hydrochloric acid or 30% lactic acid solution. Prior to adjustment, the pH of BHI + 3% HS is 7.05 while the pH of MRS is 6.16. Medium was filter-sterilized using 0.45 *μ*m filters (NALGENE, Rochester, NY, USA). Two hundred *μ*L of culture was transferred into a sterile, 96-well microplate (Corning, Inc., Corning, NY, USA). Wells containing bacteria in non-pH adjusted media and pH adjusted media without culture were used as controls. To prevent evaporation, 50 *μ*L of sterile mineral oil was pipetted gently on top of each well. Microplates were prepared anaerobically and turbidity was measured at 595 nm (Bio-Rad model 550 microplate reader, Bio-Rad Life Sciences, CA, USA) at 0, 1, 3, 6, 9, 12, 18, and 24 hours at 37°C in anaerobic conditions. This was performed twice in quadruplicate.

### 2.8. Rheological Measurements

Rheological evaluations of base gel and BPO gel formulations were measured using Hybrid Discovery HR-2 Rheometer (TA Instruments, New Castle, DE, USA) equipped with a 25 mm cross-hatched parallel steel plate and a temperature controlled parallel plate. The gap was maintained at 1.0 mm and the temperature control was set at 37°C.

Oscillation frequency sweeps and oscillation shear stress sweeps were evaluated on the following formulations: base gel and 1% BPO gel. In oscillation frequency sweeps, the shear stress was fixed at 10 pascal (Pa), within the linear viscoelastic region, and *G*′ and *G*′′ were measured between 0.5 and 20 hertz (Hz). In oscillation shear stress measurements, the frequency was fixed at 1 Hz, and shear stress increased from 10 to 1000 Pa.

Flow shear rate measurements at a variable shear rate from 0.1 to 1000/second were conducted on the formulation. After gelation, the base gel and 1% BPO gel were diluted with VFS at 25%, 50%, 75%, and 100% (gel/gel + VFS; v/v) and kept at 37°C in an incubator overnight prior to test. Viscosity using the flow shear measurements of diluted gel formulation was measured at a fixing frequency of 1 Hz. At VSF of 25% the sample was briefly stirred prior to the measurement to provide with homogenous material. All experiments were conducted in triplicate.

### 2.9. Statistics and Figure Design

All statistical analyses were performed and figures in results section were graphed in Sigma Plot 11.0. The BPO structure and breakdown were made in MarvinSketch 5.12.1.

## 3. Results and Discussion

### 3.1. Influence of BPO Gel Formulations on Microbial Growth in Well Diffusion Assay

Well diffusion assays were performed to identify the lowest concentration of BPO (w/w) at which the BV-associated pathogen *G. vaginalis* could be inhibited. Mean values and associated standard deviation of the inhibition zones are shown in Table 1. Zones of inhibition were observed in *G. vaginalis* containing plates following exposure to BPO as low as 0.01% (w/w). Zones were also confirmed in all tested higher concentrations. Zones of inhibition were observed at BPO concentrations of 2.5% or higher for all lactobacilli strains. No zones of inhibition were observed with the base gel, indicating no associated antimicrobial properties.

### 3.2. BPO Gel Formulation Selectively Inhibits *G. vaginalis* in Direct Contact Assay

**Figure 2:**
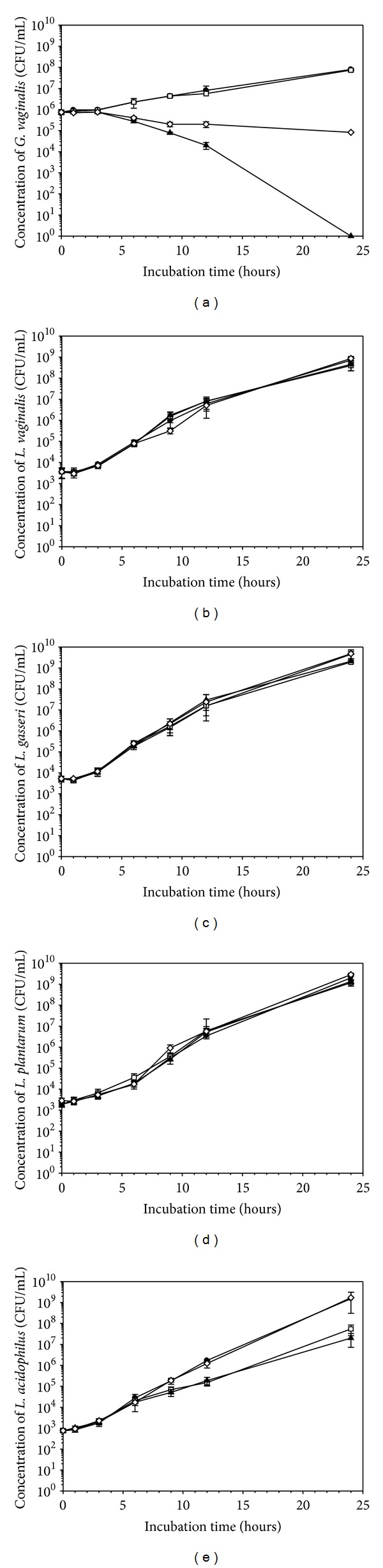
BPO inhibits *G. vaginalis* (a) but not healthy vaginal lactobacilli *L. vaginalis* (b), *L. gasseri* (c), *L. plantarum* (d), and *L. acidophilus* (e) in direct exposure experiments. (●) represents the negative control, (□) represents the base gel, (▲) represents 1% BPO hydrogel, and (*◊*) represents BPO particles. Experiments were conducted at least twice in duplicate. Mean values and standard deviations are shown.

The 1% BPO gel was chosen for these tests as it represents the highest concentration that inhibited *G. vaginalis* but had no effect on the tested *Lactobacillus* spp. in the well diffusion assay (Figures 2(a)–2(e)).

To determine survivability of *G. vaginalis*, following 24 hours of direct exposure to 1% BPO (w/w), the drop plating technique was used. Following 24 hours of exposure, a six-log reduction of the viable *G. vaginalis* cells was observed when the microorganism was grown in contact with the 1% BPO gel. *G. vaginalis* cells were not inhibited by the base gel and grew up to 7.9 × 10^7^ CFU/mL in the indirect inhibition assay or up to 7.3 × 10^7^ CFU/mL in direct contact with the base gel. Therefore, we conclude that 1% BPO gel is effective in inhibiting *G. vaginalis*. By comparison, free BPO particles were less effective than the base gel, only reducing viable cell counts by approximately three logs (Figure 2(a)).

To determine the possible effect of direct contact with BPO on the viability of vaginal *Lactobacillus* spp., the direct contact assay was repeated under the same anaerobic conditions outlined for *G. vaginalis*. Following 24 hours of incubation, no significant inhibition was noted in direct contact assays in which cultures were grown in direct contact with BPO particles, the base gel, or the 1% BPO gel (Figures 2(b)–2(d)). A two-log reduction was noted in *L. acidophilus* cultures grown in contact with the 1% BPO gel or the base gel (Figure 2(e)).

The 1% BPO hydrogel formulation is capable of inhibiting the growth of the BV-associated pathogen *G. vaginalis* while having little to no impact on the growth of selected vaginal *Lactobacillus* spp.

Continued maintenance of the vaginal ecology may improve the rate of recovery from BV. As indicated by Mitchell et al., following antibiotic treatment, vaginal lactobacilli recovery is directly proportional to cure success rate [8]. Given that most antibiotics are nonselective in nature, it is imperative to consider the continued impact on the health of the individual by establishing methods that continually maintain a stable vaginal microbiome. The hydrogel described is capable of serving such a function. It is more selective, eliminating the tested pathogenic vaginal bacterium while supporting the growth of a healthy vaginal microbiome and reducing the recurrence rate of BV without supplementary lactobacilli treatment. The suggested and described approach is a technique that is a valuable preliminary assay that addresses the complex problem of BV. Further experiments based on our study will further elucidate the interaction within polymicrobial infections and help identify methodologies to further prevent inhibition of healthy microorganisms.

### 3.3. Effect of BPO on *G. vaginalis* Viability in an “Indirect Contact” Assay

**Figure 3:**
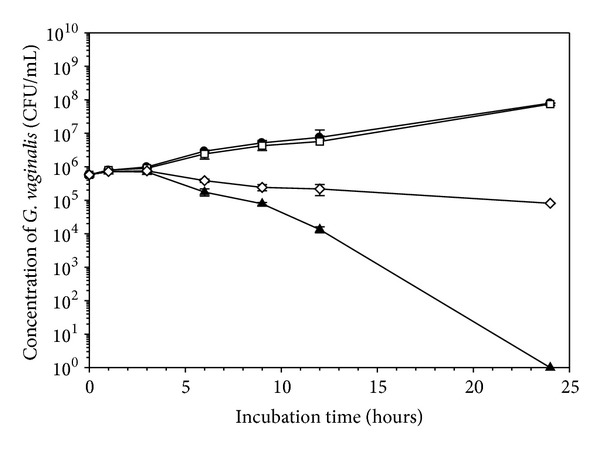
The effect of base gel, 1% BPO gel, and BPO particles on *G. vaginalis* in an indirect contact assay. All experiments were conducted twice in duplicate; (●) represents the negative control, (□) represents the base gel, (▲) represents 1% BPO hydrogel, and (*◊*) represents BPO particles. Mean values and standard deviations are shown.

Once the effect of the BPO formulations on the selected microorganisms was studied by the direct contact method, the effect in indirect exposure was evaluated using a control insert plate (Figure 3). The trials were conducted only with *G. vaginalis* at concentrations proven to be inhibitory to this vaginal pathogen because of the expense of the assay. Under conditions identical to those used in the indirect contact experiments, when exposed to gel-diffused BPO, *G. vaginalis* was inhibited similarly to what was observed in direct contact with 1% BPO gel and BPO particles (a six-log reduction in viable cell count). In this, cells were placed at the bottom of the well while the effect of placing cells on top of the inserts was not assessed.

### 3.4. Growth of Vaginal Microorganisms in pH Adjusted Media

**Figure 4:**
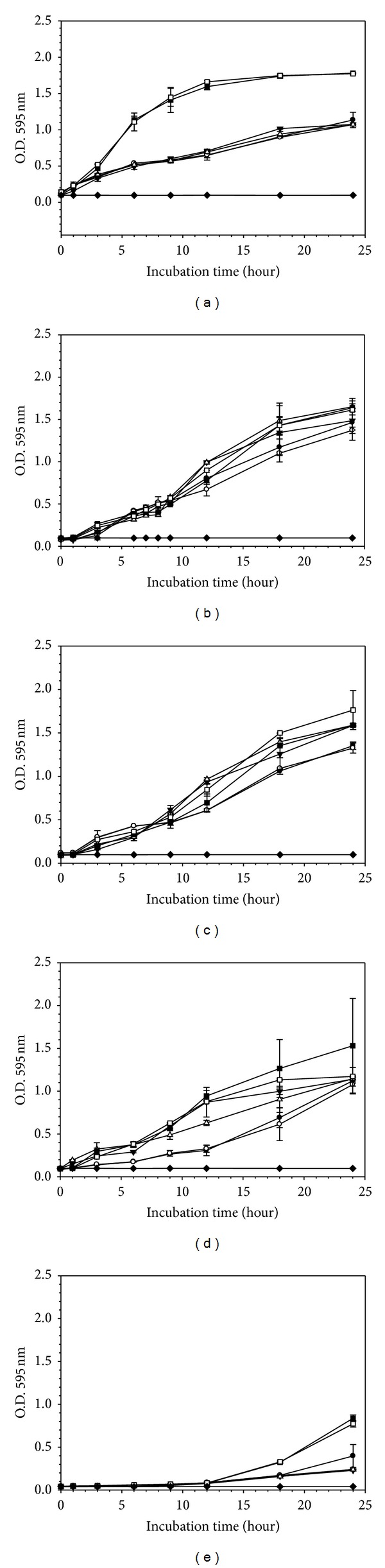
Influence of pH on growth of *G. vaginalis* (a), *L. vaginalis* (b), *L. gasseri* (c), *L. plantarum* (d), and *L. acidophilus* (e). Microbial growth was evaluated in media with pH altered to the average pH of the gels (4.5) by HCl (●, ○), lactic acid (▲, Δ), and normal growth medium (■, □) (MRS for lactobacilli spp. and BHI + 3% HS for *G. vaginalis*). Sterile broth (*◆*) was also shown as negative control. Data were collected hourly (shown only 0, 1, 3, 6, 9, 12, 18, and 24 h measurements). Experiments were conducted twice in quadruplicate.

It was observed that in the “indirect contact” experiments the base gel did not influence *G. vaginalis* viability while BPO particles caused a 3-log reduction in the number of viable cells. The base gel did however cause a 2-log reduction in *L. acidophilus* cultures. Therefore, one of the properties of the base gel (low pH of 4.5) was tested for possible effect on the growth of all tested microorganisms. *L. plantarum*, *L. gasseri,* and *L. vaginalis* grew normally while there was a slight inhibition of *L. acidophilus* and *G. vaginalis*, confirming that pH may play some role in reducing the bacterial viability (Figures 4(a)–4(e)).

### 3.5. Rheological Properties of Gel Formulations

**Figure 5:**
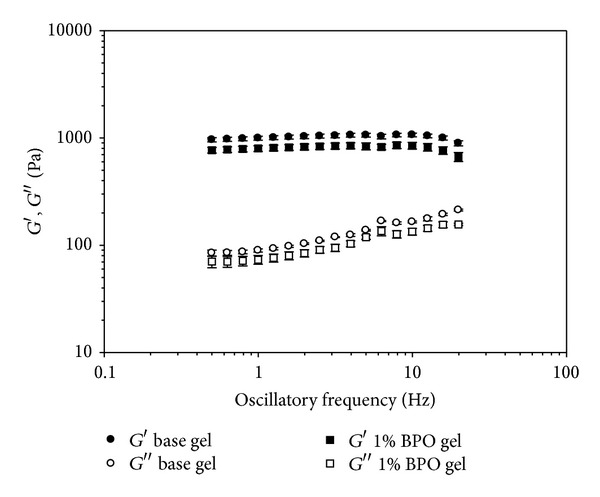
Storage modulus (*G*′) and loss modulus (*G*′′) as a function of oscillatory frequency (Hz) on the base gel and 1% BPO gel. Experiment was conducted in triplicate. Mean values and standard deviations of three experiments are shown.

The results of oscillation frequency are shown in Figure 5. In both tested formulations, from 0.5 to 20 Hz, the storage modulus (*G*′) was always greater than loss modulus (*G*′′), indicating that this material exhibits “gel-like” properties. When frequency varied from 0.5 to 20 Hz, *G*′ of base gel was significantly reduced by 1% BPO (*P* < 0.01). When frequency varied from 1.2 to 20 Hz, *G*′′ of base gel was significantly reduced by 1% BPO (*P* < 0.05). But within measured range, *G*′ of 1% BPO gel was above 600 Pa, indicating that the formulation still possesses a solid-like behavior.

**Figure 6:**
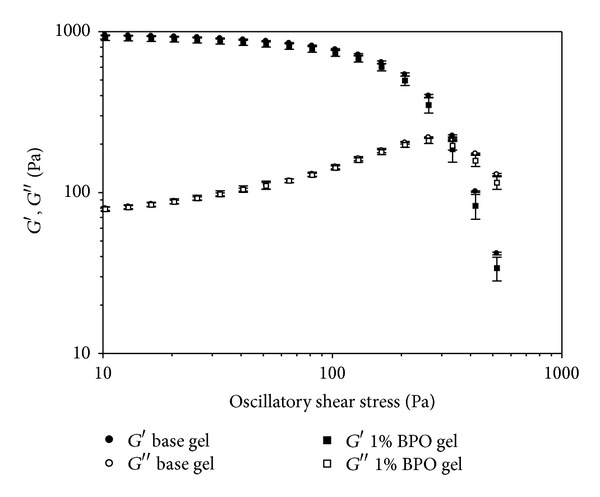
Storage modulus (*G*′) and loss modulus (*G*′′) as a function of oscillatory shear stress (Pa) on the base gel and 1% BPO gel. Experiment was conducted in triplicate. Mean values and standard deviations of three experiments are shown.

The results of oscillation shear stress are shown in Figure 6 and Table 2. Yield stress was defined as the shear stress at which its corresponding storage modulus was less than 95% of the average value from the first three detected storage moduli within the viscoelastic region, which represents initial gel elasticity. The yield stress of both formulations was approximately 30 Pa, indicating that the gel deforms upon addition of stress causing it to shear thin.

The cross-over of *G*′ and *G*′′ represents breakdown of the gel microstructure allowing the material to flow as a viscous liquid. The cross-over of *G*′ and *G*′′ of both formulations was all above 300 Pa, indicating that they possess a rigid microstructure [27].

**Figure 7:**
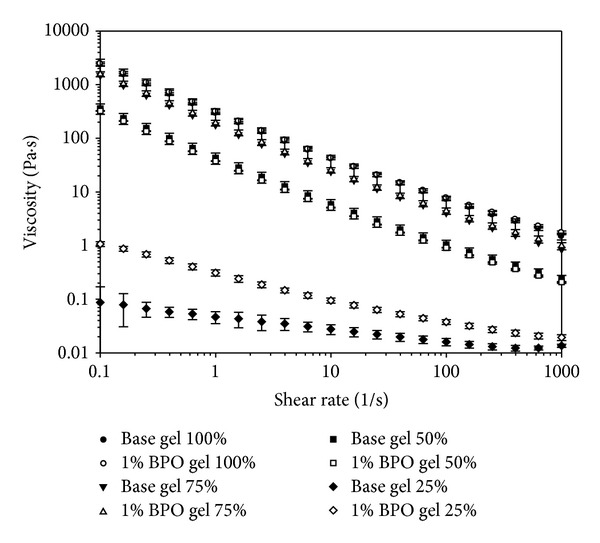
Viscosity (Pa·s) as a function of shear rate (1/s) of 100%, 75%, 50%, and 25% dilutions in VFS of base gel and 1% BPO gel. Experiment was conducted in triplicate. Mean values and standard deviations of three experiments are shown.

The results of flow shear rate are shown in Figure 7. The viscosity of all tested formulations decreased with increasing shear rate, indicating a shear thinning behavior and their injectability will be enhanced at higher shear rates. A high viscosity, at low shear rates, makes the formulations easier to stay along vagina mucosa [28]. The dispersion of BPO did not influence the viscosity of 50%, 75%, and 100% dilution of the base gel. However, 1% BPO greatly increased the viscosity of base gel when it was diluted with VFS at 25%, which could be a result from the decrease in water component from 1% BPO encapsulation. For both the base gel and the 1% BPO gel, the viscosity, as a function of shear rate ranging from 0.1 to 1000/s, decreased when the formulation was diluted with VFS, indicating that the formulation will flow more easily when diluted with vaginal fluids *in vivo*. Viscosity significantly decreased upon dilution from 50% to 25%, which may allow leakage to occur. Therefore, the presented intravaginal drug delivery system presents multiple advantages over oral drug usage in its potential ability to control BV infection. On a much larger scale, the BPO-encapsulated hydrogel conceptually mimics the pathogen-inhibiting function of healthy peroxide-producing lactobacilli. This unique antibacterial profile supports its candidacy as a viable option for treatment and, perhaps equally importantly, for prevention of recurrence of BV. Prevention of the pathogen's recurrence is crucial for effective treatment and prophylaxis of bacterial vaginosis; this can be achieved with the gel's ability to sustain healthy microbiota, thus preventing the suppressed pathogens from dominating the environment. BPO has been used in flour bleaching and acne treatment for decades. It is permitted by the FDA for use as a topical drug up to 10% as active ingredient in product treatmenting acne, based on the studies proving the safety of BPO [29]. There is no evidence which suggests that the topical application of BPO gel or lotion is directly carcinogenic [30–32]. The danger of oxidative agents when used inappropriately [33] and isolated reports on possible carcinogenic effect of BPO in animal models [34] should not be neglected. However, the commonly accepted role played by the hydrogen peroxide-producing lactobacilli in controlling vaginal pathogens [9] inspires exploration for BPO use in vaginal health maintenance. Since most current studies focus on its safety when applied topically and exposed to ultraviolet light radiation, the safety of BPO when applied intravaginally should be assessed in other models before it is approved as a BV treatment.

Future studies will include gel delivery analysis in a mouse model and identification of possible irritation of vaginal epithelium at 1% BPO, which we propose as a possible dosage level.

## 4. Conclusion

In this study, we defined and evaluated a BPO-encapsulated hydrogel formulation capable of inhibiting the growth of the BV-associated pathogen *G. vaginalis* while having a limited effect on healthy lactobacilli in the vaginal ecosystem. The rheological properties of the gel show it to be suitable for the suggested application.

## Figures and Tables

**Table 1:** Zones of inhibition (mm) from well diffusion assay^a^.

BP% (w/w)	*G. vaginalis *	*L. vaginalis *	*L. gasseri *	*L. plantarum *	*L. acidophilus *
0 (base gel)	0.00 ± 0.00	0.00 ± 0.00	0.00 ± 0.00	0.00 ± 0.00	0.00 ± 0.00
0.01	0.50 ± 0.00	0.00 ± 0.00	0.00 ± 0.00	0.00 ± 0.00	0.00 ± 0.00
0.10	0.67 ± 0.26	0.00 ± 0.00	0.00 ± 0.00	0.00 ± 0.00	0.00 ± 0.00
0.25	1.08 ± 0.20	0.00 ± 0.00	0.00 ± 0.00	0.00 ± 0.00	0.00 ± 0.00
0.50	1.17 ± 0.26	0.00 ± 0.00	0.00 ± 0.00	0.00 ± 0.00	0.00 ± 0.00
1.00	1.67 ± 0.26	0.00 ± 0.00	0.00 ± 0.00	0.00 ± 0.00	0.00 ± 0.00
2.50	1.92 ± 0.38	0.50 ± 0.00	0.50 ± 0.00	0.50 ± 0.00	0.50 ± 0.00
5.00	2.25 ± 0.42	0.50 ± 0.00	0.50 ± 0.00	0.50 ± 0.00	0.50 ± 0.26
7.50	2.42 ± 0.38	0.50 ± 0.00	0.50 ± 0.00	0.50 ± 0.00	0.58 ± 0.20
10.00	2.58 ± 0.38	0.50 ± 0.00	0.75 ± 0.27	0.50 ± 0.00	0.67 ± 0.26
Clindamycin (100 *μ*g/mL)	22.20 ± 0.80	17.30 ± 0.50	19.00 ± 0.60	21.80 ± 0.80	26.00 ± 0.90

^a^The distance is measured from the edge of the loading well to the edge of the inhibition zone in millimeters using Vernier calipers. Experiments were conducted at least two times in triplicates. Mean values and their standard deviations are provided.

**Table 2:** Yield stress and cross-over of *G*′ and *G*′′ of tested formulations^a^.

Formulation	Yield stress (Pa)	Cross-over of *G*′ and *G*′′(Pa)
Base gel	32.42 ± 1.73	345.96 ± 6.56
1% BPO gel	29.85 ± 2.72	332.58 ± 12.01

^a^Experiments were conducted in triplicate. Mean values and their standard deviations are shown.
